# Gender differences in the use of psychiatric outpatient specialist services in Tromsø, Norway are dependent on age: a population-based cross-sectional survey

**DOI:** 10.1186/s12913-015-1146-z

**Published:** 2015-10-22

**Authors:** Anne Helen Hansen, Anne Høye

**Affiliations:** Norwegian Centre for Integrated Care and Telemedicine, University Hospital of North Norway, and Faculty of Health Sciences, Department of Community Medicine, UiT-The Arctic University of Norway, PO box 35, 9038 Tromsø, Norway; Department of Psychiatric Research, University Hospital of North Norway and Center of Clinical Documentation and Evaluation (CCDE/SKDE), University Hospital of North Norway, PO box 6, 9038 Tromsø, Norway

**Keywords:** Psychiatric specialist services, Mental health care, Health care utilisation, Cross-sectional study, Norway

## Abstract

**Background:**

Overall, men are less likely than women to seek health care services for mental health problems, but differences between genders in higher age groups are equivocal. The aim of the current study was to investigate the association between gender and the use of psychiatric outpatient specialist services in Norway, both in a general population and in a subpopulation with self-reported anxiety and/or depression.

**Methods:**

Using questionnaires from 12,982 participants (30–87 years) in the cross-sectional sixth Tromsø Study (2007-8) we estimated proportions reporting anxiety/depression, and proportions using psychiatric outpatient specialist services in a year. By logistic regressions we studied the association between gender and the use of psychiatric outpatient specialist services. Analyses were adjusted for age, marital status, income, education, self-reported degree of anxiety/depression, and GP visits last year. Analyses were also performed for genders separately.

**Results:**

Anxiety/depression was reported by 21.5 % of women and 12.3 % of men in the general population. Visits to psychiatric outpatient services during one year were reported by 4.6 % of women and 3.3 % of men. The general population’s probability of a visit was significantly lower among men compared to women in ages 30–49 years (odds ratio [OR] 0.58, confidence interval [CI] 0.39–0.84, *p*-value [*p*] = 0.004), whereas men used services slightly more than women in ages 50 years and over (OR 1.36, CI 1.00–1.83, *p* = 0.047). Among those with anxiety/depression 13.5 % of women and 10.5 % of men visited psychiatric outpatient services in a year. We found no statistically significant gender differences in the use of services in this subgroup. Other factors associated with services use in women with anxiety/depression were higher education, more severe anxiety/depression, and GP visits the last year, whereas in men only a more severe anxiety/depression was associated with psychiatric outpatient visits. Overall, the use of services decreased with higher age.

**Conclusions:**

Most people with self-reported anxiety/depression did not visit specialist outpatient clinics. This applies in particular to men aged 30–49 years, older individuals, and individuals with lower education. Gender differences in the use of services in the general population were dependent on age, whereas in the subgroup with anxiety/depression gender differences were not confirmed.

## Background

Health care services and also differences in their use may contribute to inequalities in health [1, 2]. The consistent pattern in research from most high-income countries, Norway included, is that women and older people in a general population use specialist services more than men and younger people [3–5]. However, the distribution of specialist care utilisation in general populations is often studied without distinguishing somatic from psychiatric services [4, 5].

Several studies state that women in a general population use health services for mental health problems more than men [6–9], but the age distribution is equivocal. Some even suggest that older women seek more help than men [10–12], others that older men and women use services equally [13, 14]. Men and women might use services in equal proportions when controlling for disease and disease severity [15], and when taking into account that men and women differ in their reporting of mental distress levels [16].

Mental disorders are the leading global cause of all non-fatal burden of disease, with depressive and anxiety disorders accounting for most of that burden [17]. Still, around half of depressed persons worldwide do not receive treatment [18]. Since most mental disorders emerge before the age of 30 [19, 20], lack of proper treatment might contribute to disability regarding family life, employment and leisure activities for many crucial years of an individual’s life. In Norway, anxiety and depression are estimated with a 12 months prevalence around 15 % and 10 %, respectively [21]. Lifetime and 12 months prevalence are estimated to be around twice as high in women as in men [21]. The use of mental health services is affected by treatment provisions, public assessments of acceptability, accessibility, and usefulness of services. These factors will vary between different population groups, between countries, and over time. It is, for instance, well known that male suicide rates in the Nordic countries are consistently higher than female rates [22]. Hence, research from different health systems in different cultural settings is important to achieve a comprehensive epidemiological view, and to identify vulnerable subgroups. Recent research in this field has largely been lacking in Norway despite the volume and disability of anxiety/depression in the population, but is needed to help communities shape psychiatric services according to population needs.

In Norway all citizens are provided a regular GP, and only 0,4 % of the population has chosen to remain outside GPs’ lists [23]. First-line medical services including emergency clinics are run by the municipalities. Specialist services, consisting of hospitals and outpatient clinics, are run by regional health enterprises mainly owned by the state. Access to specialist care is usually achieved by referral from the GP (the gate-keeper role). Norway has universal insurance, and GP and specialist outpatient visits are co-payed by a small fee.

Tromsø is the largest city in North Norway with a population of 65,286 (January 2008). The municipality is almost equal to Norway in terms of key parameters including employment and unemployment, average gross income per capita, proportion of disability pensioners, number of physicians per 10,000 residents, and proportion of the population living in urban areas. However, the population is younger and higher educated than the Norwegian average [24]. Tromsø hosts the University Hospital of North Norway with somatic and psychiatric services.

The aim of the current study was to investigate whether the use of psychiatric outpatient specialist services was associated with gender in a population-based cohort, and in a subpopulation with self-reported anxiety and/or depression in Tromsø, Norway. Our hypothesis was that women in the general population used services more than men, but that this gender difference was not present in the group with self-reported anxiety and/or depression.

## Methods

### Study population

Population-based health surveys have been conducted in Tromsø since 1974 [25, 26]. The cross-sectional sixth Tromsø Study (Tromsø 6), consisting of two comprehensive self-administered questionnaires, clinical examinations and laboratory tests, was conducted from October 2007 to December 2008. Four groups were invited; every resident aged 40–42 or 60–87 years (*n* = 12,578), a 10 % random sample of individuals aged 30–39 (*n* = 1,056), a 40 % random sample of people aged 43–59 (*n* = 5,787) and all subjects who had attended the second visit of the fourth Tromsø Study, if not already included in the other three groups (*n* = 341). The sampling reflected the need for repeated measurements and follow-up, and the need to enrol new participants for new and ongoing projects.

Our data was collected from the two self-administered questionnaires. The first questionnaire was mailed with the invitation about two weeks ahead of the suggested appointment time. Participants were invited to attend whenever suitable within the survey opening hours (between 09:00 and 18:00). Non-respondents were given one reminder. The second questionnaire was handed out upon attendance, and most participants completed it while waiting for the clinical examination. The comprehensive Tromsø 6 data include self-reported demographic and socio-economic characteristics, information about symptoms and diseases, health status, and use of medicines and health services. Since residents with severe mental disorders are unlikely to participate in population-based surveys like Tromsø 6 [27] the study mainly includes persons without psychotic symptomatology.

**Fig. 1:**
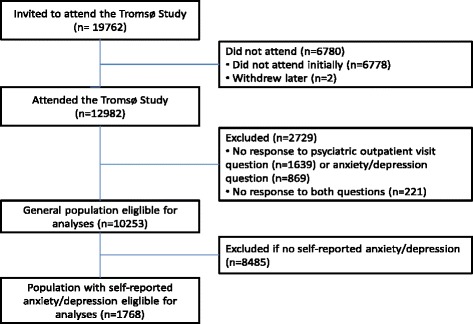
Flow chart of study population

In the present study we excluded participants who failed to inform about psychiatric outpatient visits (*n* = 1,639), anxiety/depression (*n* = 869), or both (*n* = 221). The final general population sample consisted of 10,253 participants (Fig. 1). For further analyses of participants with moderate or severe anxiety/depression we excluded those who reported no anxiety/depression (*n* = 8,485). This final sample consisted of 1,768 participants (Fig. 1).

### Measures

The use of psychiatric outpatient services at least once during the previous year (yes/no) was designated as the dependent variable. This included visits to private and public providers, and also visits to hospital staff like nurses and social workers, supervised by psychiatrists and psychologists.

Gender was designated key independent variable. Adjustment independent variables were age in 10-year groups, marital status, income, education, self-reported degree of anxiety/depression, and GP visits last year (yes/no). For marital status we used the original response options: married/cohabitant or single. The income variable referred to the household’s total gross income in the previous year. Eight original response categories were merged into low income (< NOK 200,000), low middle income (NOK 201,000-400,000), high middle income (NOK 401,000-700,000) and high income (> NOK 700,000). We defined three education response categories from the original five: low (primary and part of secondary school), middle (high school) and high education (college or university). The self-reported anxiety/depression variable was obtained from the Euro Quality of Life Group five Dimensions (EQ-5D) score question with the three response options “I am not/moderately/extremely anxious/depressed” [28].

### Analyses

Data was analysed by means of descriptive statistics and logistic regressions. Differences between men and women were investigated by applying univariable as well as multivariable logistic regression models adjusted for age, marital status, income, education, self-reported degree of anxiety/depression, and GP visits last year. Analyses were performed for both genders as well as for men and women separately. The adjustment variables were introduced collectively into the regression models. All analyses were obtained as dummy- and trend analyses. Since some of the groups in the anxiety/depression sample were small, and there was no significant lack of linearity, we chose to report the trend analyses exclusively. First-order interactions were tested by introducing interaction terms in the regression models, and by stratifications.

We used 95 % confidence intervals (CI)/*p* < 0.05 as significance level throughout the study. All analyses were accomplished using Stata, version 13.1.

### Ethics

The sixth Tromsø Study and this particular study have been approved by the Regional Committee for Medical and Health Research Ethics (REK 2009/2536 and 2014/1667/REK nord).

## Results

In total 12,982 persons aged 30–87 years participated in Tromsø 6, constituting an overall response rate of 65.7 % [25]. More women than men participated in the study, and more women lived in lower income and single person households (Table 1). Persons with high education, high middle income, no anxiety/depression, one or more GP visits last year, and those living with a spouse made up the largest groups in both genders. The same applied to the subsample with self-reported anxiety/depression, except that the largest group among women had low middle income and low education, whereas among men the largest group had high middle income and high education (Table 1). In both genders a moderate degree of self-reported anxiety/depression was far more common than a severe anxiety/depression (Table 1).

**Table 1:** Characteristics of the general sample and of the sample with self-reported anxiety or depression (%)

	General population			Population with anxiety/depression		
	Both genders	Women (51.7 %)	Men (48.3 %)	Both genders	Women (64.5 %)	Men (35.5 %)
Psychiatric outpatient visits last year	*n* = 10253	*n* = 5304	*n* = 4949	*n* = 1768	*n* = 1140	*n* = 628
No	96.1	95.4	96.7	87.6	86.5	89.5
Yes	3.9	4.6	3.3	12.4	13.5	10.5
Age	*n* = 10253	*n* = 5304	*n* = 4949	*n* = 1768	*n* = 1140	*n* = 628
30–39	4.1	4.5	3.7	4.5	4.8	3.8
40–49	29.9	30.5	29.3	29.0	27.1	32.2
50–59	19.6	19.5	19.6	21.4	21.1	22.0
60–69	30.9	29.7	32.1	27.3	26.8	28.0
70–79	12.4	12.3	12.6	14.1	15.3	11.9
80–87	3.1	3.5	2.7	3.7	4.7	1.9
Marital status	*n* = 10010	*n* = 5138	*n* = 4872	*n* = 1719	*n* = 1100	*n* = 619
Single	23.6	29.7	17.1	32.5	36.4	25.7
Married/cohabitant	76.4	70.3	82.9	67.5	63.6	74.3
Household income^a^	*n* = 9625	*n* = 4858	*n* = 4767	*n* = 1640	*n* = 1033	*n* = 607
Low	10.2	13.5	6.7	17.9	21.6	11.5
Low middle	25.8	28.5	23.1	32.1	32.8	31.0
High middle	36.3	33.1	39.5	32.4	30.2	36.1
High	27.7	24.9	30.7	17.6	15.3	21.4
Education^b^	*n* = 10149	*n* = 5249	*n* = 4900	*n* = 1740	*n* = 1126	*n* = 614
Low	26.2	28.9	23.4	33.7	35.8	29.8
Middle	33.6	31.9	35.3	32.2	32.8	31.3
High	40.2	39.2	41.3	34.1	31.4	38.9
Degree of anxiety/depression	*n* = 10253	*n* = 5304	*n* = 4949	*n* = 1768	*n* = 1140	*n* = 628
Not at all	82.8	78.5	87.3	-	-	-
Moderate	16.8	20.9	12.4	97.3	97.0	97.9
Severe	0.5	0.6	0.3	2.7	3.0	2.1
GP visits last year	*n* = 10197	*n* = 5269	*n* = 4928	*n* = 1763	*n* = 1136	*n* = 627
No	19.3	15.4	23.5	9.5	7.9	12.4
Yes	80.7	84.6	76.5	90.5	92.1	87.6

*GP* general practitioner

^a^Low (<200000 NOK), Low middle (201000–400000 NOK), High middle (401000–700000 NOK), High (>700000 NOK)

^b^Low (primary/part of secondary school), Middle (high school), High (college/university)

### General population

Table 1 gives the characteristics of the general population and the population with anxiety/depression. In total, 17.3 % of the general population reported anxiety/depression, 21.5 % of women and 12.3 % of men. Visits to psychiatric outpatient services during a year were reported by 3.9 %, 4.6 % among women and 3.3 % among men (Table 1).

**Fig. 2:**
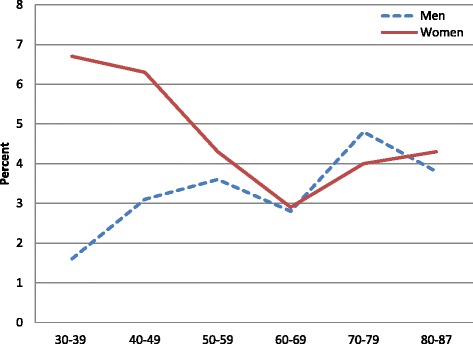
Proportion of the general population visiting psychiatric outpatient services once or more in a year, by gender and age

Univarable logistic regressions showed that the likelihood of visiting psychiatric outpatient services was lower among men than women (OR 0.71, CI 0.58–0.87, *p* = 0.001). These gender differences were not maintained after adjustment for age, marital status, income, education, self-reported degree of anxiety/depression, and GP visits last year (Table 2), thus confirming our hypothesis. The relation between the use of services and gender was, however, modified by age, with an increased probability of use among women compared to men in younger ages (interaction term gender x age OR 1.54, CI 1.27–1.87, *p* < 0.001). Analyses stratified by age confirmed that men used psychiatric outpatient services significantly less than women in ages 30–49 years (OR 0.58, CI 0.39–0.84, *p* = 0.004), but slightly more than women in ages 50 years and over (OR 1.36, CI 1.00–1.83, *p* = 0.047). The likelihood of a visit to psychiatric outpatient services decreased by age in women aged 30–69 years, whereas it increased by age in men aged 30–59 years (Fig. 2). Overall, the use of services decreased significantly with higher age (Table 2).

**Table 2:** Probability of psychiatric outpatient visits once or more during the previous year in a general population (multivariable logistic regressions)

	**Both genders** ***n*** **= 9303**		**Women** ***n*** **= 4659**		**Men** ***n*** **= 4644**	
	OR for trend	95 % CI (*p*-value)	OR for trend	95 % CI (*p*-value)	OR for trend	95 % CI (*p*-value)
**Gender** ^**a**^	0.98	0.78–1.23 (0.855)	**-**	**-**	**-**	**-**
**Age in 10 year groups**	**0.82**	**0.74–0.91 (<0.001)**	**0.69**	**0.60–0.80 (<0.001)**	1.05	0.90–1.23 (0.536)
**Marital status** ^**b**^	0.89	0.67–1.18 (0.415)	0.82	0.56–1.09 (0.299)	0.87	0.55–1.38 (0.545)
**Household income** ^**c**^	**0.79**	**0.67–0.93 (0.004)**	**0.75**	**0.61–0.93 (0.009)**	0.89	0.69–1.14 (0.346)
**Education** ^**d**^	**1.33**	**1.13–1.57 (<0.001)**	**1.53**	**1.22–1.91 (<0.001)**	1.04	0.82–1.32 (0.716)
**Degree of anxiety/depression** ^**e**^	**5.35**	**4.35–6.58 (<0.001)**	**6.50**	**4.93–8.58 (<0.001)**	**4.50**	**3.23–6.26 (<0.001)**
**GP visits last year** ^**f**^	**3.98**	**2.42–6.53 (<0.001)**	**4.56**	**2.12–9.84 (<0.001)**	**3.35**	**1.74–6.45 (<0.001)**

*OR* odds ratio, *CI* confidence interval

Statistically significant findings (95 % CI/*p* < 0.05) are marked in bold

^a^Gender: 0 = women, 1 = men

^b^Marital status: 0 = single, 1 = married/cohabitant

^c^Household income: 1 = low, 2 = low middle, 3 = high middle, 4 = high

^d^Education: 1 = low, 2 = middle, 3 = high

^e^Degree of anxiety or depression: 1 = not at all, 2 = moderate, 3 = severe

^f^GP visits last year: 0 = no, 1 = yes

Furthermore, there was a statistically significant reduced likelihood of visits among men compared to women as education increased (interaction term gender x education OR 0.65, CI 0.49–0.87, *p* = 0.003), meaning that highly educated women used psychiatric services relatively more than men, and that the gender difference increased with higher education.

Other factors associated with the use of psychiatric outpatient services in the general population were lower income, higher education, more severe anxiety/depression, and GP visits the last year (Table 2). We found the same for women when analysing genders separately, whereas for men anxiety/depression and GP visits during the last year were the only statistically significant variables associated with use (Table 2).

### Population with anxiety/depression

**Fig. 3:**
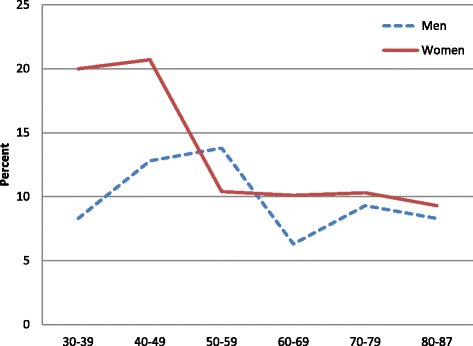
Proportion of participants with self-reported anxiety/depression visiting psychiatric outpatient services once or more in a year, by gender and age

The likelihood of visiting psychiatric outpatient services decreased with age in women with anxiety/depression, whereas men’s probability of visiting peaked in ages 40-59 years (Fig. 3). Only 12.4 % with anxiety/depression reported psychiatric outpatient visits during the last year, 13.5 % of women and 10.5 % of men (Table 1). Among those with severe anxiety/depression, 52.9 % of women and 30 % of men reported visits. Corresponding numbers among those with moderate anxiety/depression were 12.3 % and 10.1 %, respectively.

In line with our hypothesis, there were no statistically significant gender difference among those with self-reported anxiety/depression; neither in univariable analysis (OR 0.75, CI 0.55–1.02, *p* = 0.068) nor in multivariable analysis adjusted for age, marital status, income, education, self-reported degree of anxiety/depression, and GP visits last year (Table 3). Use was significantly reduced by higher age (Table 3).

**Table 3:** Probability of psychiatric outpatient visits once or more during the previous year in a population with self-reported anxiety/depression (multivariable logistic regressions)

	**Both genders** ***n*** **= 1575**		**Women** ***n*** **= 987**		**Men** ***n*** **= 588**	
	OR for trend	95 % CI (*p*-value)	OR for trend	95 % CI (*p*-value)	OR for trend	95 % CI (*p*-value)
**Gender** ^**a**^	0.76	0.54–1.06 (0.109)	**-**	**-**	**-**	**-**
**Age in 10 year groups**	**0.73**	**0.63–0.85 (<0.001)**	**0.71**	**0.60–0.85 (<0.001)**	0.79	0.61–1.02 (0.074)
**Marital status** ^**b**^	0.88	0.59–1.32 (0.544)	0.92	0.57–1.49 (0.732)	0.79	0.38–1.63 (0.525)
**Household Income** ^**c**^	0.91	0.73–1.14 (0.418)	0.89	0.68–1.16 (0.389)	0.98	0.67–1.44 (0.918)
**Education** ^**d**^	**1.34**	**1.08–1.68 (0.009)**	**1.42**	**1.08–1.89 (0.014)**	1.21	0.84–1.75 (0.316)
**Degree of anxiety/depression** ^**e**^	**7.96**	**3.96–16.00 (<0.001)**	**9.85**	**4.21–23.04 (<0.001)**	**5.04**	**1.38–18.44 (0.014)**
**GP visits last year** ^**f**^	**2.92**	**1.40–6.09 (0.004)**	**3.54**	**1.26–9.98 (0.017)**	2.29	0.80–6.57 (0.124)

*OR* odds ratio, *CI* confidence interval

Statistically significant findings (95 % CI/*p* < 0.05) are marked in bold

^a^Gender: 0 = women, 1 = men

^b^Marital status: 0 = single, 1 = married/cohabitant

^c^Household income: 1 = low, 2 = low middle, 3 = high middle, 4 = high

^d^Education: 1 = low, 2 = middle, 3 = high

^e^Degree of anxiety or depression: 2 = moderate, 3 = severe

^f^GP visits last year: 0 = no, 1 = yes

Among women with anxiety/depression we found younger age, higher education, more severe degree of anxiety/depression, and GP visits the last year to be associated with use, whereas in men only a more severe anxiety/depression was associated with psychiatric outpatient visits (Table 3).

## Discussion

### Key findings

The main finding of the current study is that in a general population the probability of a psychiatric outpatient visit was significantly lower among men compared to women in ages 30–49 years, whereas the trend was the opposite for ages 50 years and over. Only 3.9 % of the general population visited psychiatric outpatient services in a year. In both genders the probability of visiting was strongly associated with symptoms of anxiety/depression, and GP visits during the last year. In women the likelihood of a visit was also associated with lower age, lower income, and higher education.

### Comparison with other studies

Among those with self-reported anxiety/depression only12.4 % visited psychiatric outpatient services in a year. There were no statistically significant gender differences in use of services in this subgroup. The probability of visiting was strongly associated with more severe symptoms of anxiety/depression. In women the likelihood of a visit was additionally associated with higher education and GP visits the last year.

It is well documented throughout the world that men use mental health services less than women [29–32], but the picture has been ambiguous for higher age groups [10–14]. Our finding that gender differences in psychiatric outpatient specialist utilisation are dependent on age in a general population has, to our knowledge, hardly been reported in previous studies. Olivier et al. found that men in a general GP population in Somerset England were less likely to say that they would seek any kind of help for mental health problems, and that help-seeking increased steeper by age in men compared to women [33]. This does not underpin our finding that women’s use of psychiatric specialists decreased by age, but substantiates our study regarding the relative use between genders since utilisation rates for men and women approached in higher ages. In line with our study, Mosier et al. found that Canadian women’s probability of service use for mental health reasons decreased with age [30]. We found no significant trend in men’s utilisation by age, whereas Mosier et al. found an age dependent increase in men’s service utilisation [16, 30]. There is, however, evidence that the prevalence of anxiety and depressive disorders decrease by age throughout a lifespan both in men and women [19, 30, 34], strongly indicating that other factors than prevalence of disease are of great importance in understanding patterns of psychiatric services utilisation, particularly in men. Possible explanations for the opposite gender differences in ages 30-49 and 50 years and over could be that men might not recognize and accept mental health problems to the same extent as women during their 30s and 40s [35], which in turn may relate to requirements for social manifestations of masculinity in a traditional sense [36–38]. These notions might be intertwined with the phenomenon of stigma, leading to patients preferring not to receive care at all or not to receive care in specialist psychiatric settings [10, 39, 40]. Pattyn et al. found that men were more likely than women to be recommended self-care for mental health problems, both by women and by other men. This report also states that men report more “negative attitudes towards professional help-seeking, but that also women (re)construct masculinity norms by giving different treatment advice to a man compared to another woman” [40]. This might suggest that men should be encouraged to perceive, recognize and accept mental problems to a larger extent, and that the acceptance of such problems among men, in particular among younger men (<50 years), should increase in society and health services.

In the subpopulation with self-reported anxiety/depression we found no statistically significant gender differences in use of outpatient psychiatric services. This is in line with previous studies reporting that genders do not differ in use of services once mental problems are perceived and recognised [9, 15, 16, 30, 35].

Our finding that use was generally lower in higher ages (Tables 2 and 3) is in accordance with studies from Norway [41] and other countries [7, 10, 12, 42, 43]. It is consistently reported that older adults (55–65 years and over) of both genders are less likely than younger and middle aged to perceive a need for care, to receive referrals from primary care, and to use specialist psychiatric services [10, 43]. In older adults visits to the GP for mental health problems seem to be more common than specialist visits [13]. This might implicate that health care providers and services should be aware of a possible under-treatment of older adults compared to younger adults when treatment in specialist services is concerned.

Only 12.4 % of those who reported some degree of anxiety/depression had visited psychiatric specialist services during the previous year. Wang et al. found that among patients with mood, anxiety and/or substance disorders in 10 different high-income countries 37.6 % (New Zealand) to 52.2 % (Spain) received specialist care [18]. We found that even among patients with severe symptoms less than half (46.8 %) had visited specialist services, whereas Wang et al. found that 24 % (Japan) to 61 % (Belgium) had visited specialists. A Canadian survey showed that 54 % of respondents meeting criteria for major depression had consulted health care services for mental health reasons in the previous year (with no significant differences between genders) [9]. Hämäläinen et al. even reported that about half of those suffering from severe anxiety/depression in Finland did not use any health care services for these symptoms [42], whereas specialist level services accounted for 64 % of use associated with major depressive disorders, and 71 % of use associated with anxiety disorders [39]. It has been estimated that around half of those who use services for mental health problems in high-income countries use specialty services [18]. Thus, it is likely that some of our study participants, who did not visit psychiatric specialist services, received GP treatment for their mental health symptoms. However, Olivier et al. found that the preferred source of help for mental health problems was relatives and friends, and that one in four said that they would not seek help anywhere [33]. One in six would not seek help from their GP [33]. Our low visit rates adds to a solid documentation that the use of mental health services both in general populations and in people with depression and anxiety disorders in high income countries is limited, indicating that these symptoms are undertreated [18, 34, 44]. Treatment rates are even lower in low and middle income countries [18].

In line with others’ research, our study shows that the main determinant for seeking help for mental health problems was the severity of the symptoms [8, 33, 35, 42, 45]. Furthermore, it is not surprising that GP visits also turned out to be an important indicator of specialist utilisation in both genders, since referrals are required for specialist treatment in Norway.

### Association with socio-economic status

The likelihood of psychiatric specialist use in a general population increased with higher education but decreased with higher income. This finding is in accordance with research from Norway [46] and the US [7], and has been discussed elsewhere [3]. However, we revealed an effect modification for gender and education, indicating even higher use among women compared to men as education increases. Schomerus et al. reported an association of higher education and help-seeking for depression, but did not observe any gender-specific effect [47]. Our finding might be due to an increased perception of mental problems in highly educated women combined with knowledge that effective treatment exists, and a larger willingness to seek care compared to highly educated men.

### Strengths and limitations

Particular strengths of this study were the large sample size, the high response rate, and the comprehensive coverage of information about health, disease, and socio-economic status in the questionnaires.

Nevertheless, the study should be interpreted in light of some limitations. Despite a high response rate, our sample may not be entirely representative of the general population, as it is well known that women, married/cohabitants, healthier persons, and higher socio-economic groups are more likely to participate in population surveys [48]. In Tromsø 6, attendees were older, and the proportions of married/cohabitants and women were higher than in non-attendees [25, 26]. In the second Tromsø Study (1979–80) the participation of men and women with psychiatric morbidity was approximately 20 % lower than for those without such morbidity [27], and lower participation is likely the case for Tromsø 6 as well. However, this applies particularly to serious psychiatric morbidity [27, 49]. Additionally, our data might underestimate psychiatric morbidity and treatment seeking due to perceived stigma [10], since the numbers failing to inform about psychiatric visits and/or morbidity were relatively high. The validity of symptoms scored by the EQ-5D may be disputable, although EQ-5D has been shown to achieve adequate levels of performance in depression and to some extent in anxiety [50]. It is thus possible that these symptoms are underestimated in our population. Our data might also underestimate treatment seeking since questions about psychiatric conditions and use of services were spread throughout the questionnaire, potentially increasing inaccuracies [51]. However, there is hardly any reason why people should report anxiety/depression but not use of psychiatric services, thus the relative validity between these variables should be quite robust. The validity of self-reported data as such may be questioned, although agreement between self-reported and registered health care utilisation is generally high [52]. It might also be easier to report anxiety/depression in a self-administered questionnaire than reporting to health care providers. Therefore, self-reported anxiety/depression might be the best available measure for our study purpose, since research based on doctor made diagnoses would make it difficult to include those who had not visited health care services. In our analyses we focused on anxiety/depression, but we cannot rule out the possibility that participants may have had other psychiatric ailments or diseases in addition. Furthermore, it might be a problem that we asked about anxiety/depression at the time of the survey, whereas health care utilisation was reported for the previous 12 months. However, the onset of these diseases is often ahead of 30 years of age [19, 20], making it unlikely that this have affected our study. Finally, we cannot exclude the possibility of unmeasured confounders of the reported associations.

## Conclusions

Even in a public health system like the Norwegian, few people with self-reported anxiety/depression visit specialist outpatient clinics. This applies in particular to men aged 30-49 years, older individuals, and individuals with lower education. Gender differences in the use of services in the general population were dependent on age, whereas in the subgroup with anxiety/depression gender differences were not confirmed. Identification of individuals and vulnerable subgroups in need of treatment is highly important to ensure equality in health care. Together with international documentation, our study indicates that anxiety/depression might be undertreated. In light of the burden of these diseases, improvement of population health might do a big step forward if prevention and proper treatment of mental illness is made a real public health priority.

## Abbreviations

CI: Confidence Interval
EQ-5D: Euro Quality of Life Group five Dimensions score scale
GP: General Practitioner
NOK: Norwegian Kroner
OR: Odds ratio
p: *P*-value
Tromsø 6: The sixth Tromsø Study
US: United States of America
